# 24 h mortality and its predictors among road traffic accident victims in a resource limited setting; a multicenter cohort study

**DOI:** 10.1186/s12893-023-02011-9

**Published:** 2023-04-26

**Authors:** Kinyamaniyi Kamabu, Jorge La O Soria, Deus Tumwesigye, Xaviour Francis Okedi, Lauben Kyomukama, Joshua Muhumuza, Brian Musinguzi, Daniel Kavuma, Bives Mutume Nzanzu Vivalya, Michael Loduk, Wani Shabani Abdullah

**Affiliations:** 1 Department of Surgery, Faculty of Clinical Medicine and Dentistry, Kampala International University Western Campus, PO. Box 70, Ishaka-Bushenyi, Uganda; 2 Department of Surgery, Mbarara University of Science and Technology, Mbarara, Uganda; 3 Department of Psychiatry, Faculty of Clinical Medicine and Dentistry, Kampala International University Western Campus, Ishaka-Bushenyi, Uganda

**Keywords:** 24 h mortality, Road traffic accident, Low income country, Predictors

## Abstract

**Introduction:**

The incidence of road traffic accidents (RTAs) is on the rise contributing to the global burden of mortality as a major global health threat. It has been estimated that 93% of RTAs and more than 90% of the resulting deaths occur in low and middle income countries. Though death due to RTAs has been occurring at an alarming rate, there is paucity of data relating to incidence and predictors of early mortality. This study was aimed at determining the 24 h mortality and its predictors among RTA patients attending selected hospitals in western Uganda.

**Methods:**

This was a prospective cohort that consecutively enrolled 211 RTA victims admitted and managed in emergency units of 6 hospitals in western Uganda. All patients who presented with a history of trauma were managed according to the advanced trauma life support protocol (ATLS). The outcome regarding death was documented at 24 h from injury. Data was analyzed using SPSS version 22 for windows.

**Results:**

Majority of the participants were male (85.8%) aged 15–45 years (76.3%). The most common road user category was motorcyclists (48.8%). The 24 h mortality was 14.69%. At multivariate analysis, it was observed that a motorcyclist was 5.917 times more likely to die compared to a pedestrian (*P* = 0.016). It was also observed that a patient with severe injury was 15.625 times more likely to die compared to one with a moderate injury (*P* < 0.001).

**Conclusion:**

The incidence of 24 h mortality among road traffic accident victims was high. Being motorcycle rider and severity of injury according to Kampala trauma score II predicted mortality. Motorcyclists should be reminded to be more careful while using the road. Trauma patients should be assessed for severity, and the findings used to guide management since severity predicted mortality.

## Background

The beginning of the road traffic accident (RTA) problem started before the introduction of cars; but the problem intensified with the invention of vehicles. According to the world health organization fact sheets published in June 2022 [1], about 1.3 million people succumb to road traffic accidents each year, with 93% of the deaths occurring in Low and Middle Income Countries (LMICs). Furthermore a recent evaluation of WHO trends [2] predicted RTAs to be the seventh main cause of deaths by 2030 unless action is taken. Thus, the incidence of RTA is on the rise contributing to the global burden of mortality as a major global health threat [3].

According to Jayaraman et al. at Mulago National Referral Hospital, death due to RTAs accounted for half of the total deaths with majority dying within 24 h. Over the last 25 years, RTA related deaths in Uganda have risen seven-fold with an accident severity index of 24 deaths per 100 road crashes, an average of 10 people dying per day, which is the highest rate in the East African community [4, 5]. Proper and timely management of trauma patients is thought to be a common dilemma in which the accuracy of fulfilling the requirements of this management is very difficult in some of our settings that lack provisions and expertise to handle RTA emergencies [6]. This always results in delayed or no care to trauma patients which eventually may contribute to their death.

While death due to road traffic injury has been occurring at an alarming rate as reported in many studies [7], few of these surveys have highlighted in-hospital incidence and predictors of early death. As majority of deaths due to RTAs are largely preventable [2], all sectors, including health, need to be fully engaged in the responsibility. Therefore, identification of the predictors of early mortality will not only help to build comprehensive management strategies but also help reduce preventable mortality due to RTAs in Ugandan Hospitals. This study was aimed at determining the 24 h mortality and its predictors among RTA patients attending selected hospitals in western Uganda.

## Materials and methods

### Study design

This was a prospective cohort that enrolled road traffic accident (RTA) victims admitted and managed in emergency units of 6 hospitals in western Uganda. The prospective cohort design was chosen to enable follow up of the patients and identify mortality.

### Study setting

The study centres included 1 regional referral hospital (Mbarara Regional Referral Hospital (MRRH)), 1 university teaching hospital (Kampala International University-Teaching Hospital (KIU-TH)) and 4 general hospitals (Ishaka Adventist Hospital(IAH), Bwera General Hospital (BGH), Comboni Hospital (CH) and Kilembe Mines Hospital (KMH)). All the above hospitals provide fulltime emergency trauma services. Many trauma patients are admitted in these facilities and managed. These study centres were chosen because they represent all categories of hospitals usually encountered in Uganda (regional referral hospitals, teaching hospitals, government hospitals and private hospitals). This would give a representative picture of hospitals in Uganda.

### Sample size determination and sampling

Using Daniel’s formula [8] and the findings from Oporia et al. study [9] that reported a mortality of 14.61% among trauma patients in Uganda, the required sample size was determined to be 192. $n=\frac{{Z}^{2}\left(P\left(1-P\right)\right)}{{d}^{2}}=\frac{{1.96}^{2}\left(0.146\left(1-0.146\right)\right)}{{0.05}^{2}}\cong 192$. On adding 10% to increase internal validity, 211 participants were required. Convenience sampling method was used in which all eligible road traffic accident victims were enrolled consecutively till the sample size required was reached.

### Inclusion and exclusion criteria

All patients with history of RTA admitted in the emergency units of the selected hospitals were included after providing informed consent. Unconscious patients were also included after obtaining consent from a legal guardian. Patients who presented 24 h after injury were excluded.

### Data collection procedure

All patients who presented with a history of trauma were managed according to the advanced trauma life support protocol (ATLS) [10]. Filling of the questionnaire started at arrival and the questionnaire was updated during follow up till 24 h when the outcome was documented. At presentation, sociodemographic characteristics of the patient, category of road user, mechanism of injury and time of accident were documented as narrated by the patient or the patient attendant who witnessed the accident. Findings of the assessment of the patient including time of the first aid, severity of injury using Kampala trauma score II (KTS II) [11], type of injury, site of injury, treatment provided and any change during 24 h were documented. The Kampala trauma score II (KTS II) was determined based on the age, systolic blood pressure, respiratory rate, neurological status, and score for serious injury with a minimum score of 0 and a maximum score of 10 [11]. The lower the score, the more severe the injury [11]. This score has been validated for use in Uganda and found to be comparable to the injury severity score and the new injury severity score in predicting mortality [11]. The outcome regarding death was documented at 24 h from injury. To those who did not know English, a translated form of the questionnaire was used. To ensure data completeness, we crosschecked the questionnaires just after filling, and any missing information was added. To ensure quality of the data, the inclusion and exclusion criteria were strictly adhered to. We were focused on the inclusion and exclusion criteria and we were following the standardized questionnaire for all participants. Also the questionnaire was pre tested for validity and reliability before starting data collection and appropriate adjustments made. Data collection lasted 3 months. The data collection was done by qualified doctors with a minimum of a bachelor’s degree in medicine and surgery that attended to the patients in the accident and emergency units at the study centres.

### Study variables

The independent variables included pre-hospital and in-hospital characteristics. The pre-hospital characteristics included: time interval from accident to hospital, age, road user category, mechanism of injury and comorbidity. In-hospital characteristics included: time interval from arrival at hospital to attaining medical care, cadre of the first health worker that attended to the patient, KTS II severity score, type of injury, site of injury and treatment given. The dependent variable was mortality defined as death or no death.

### Data analysis

Data was analyzed using SPSS version 22 for windows. The mortality was computed as the number of patients that died divided by the total number of trauma patients included in the study. To determine the predictors of mortality, bivariate binary logistic regression was done. Those variables that were significant at the bivariate level, were re analyzed using multivariate back ward stepwise regression. The corresponding adjusted odds ratios, *p* values and 95% confidence intervals were reported. Variables that appeared in the final model with a *p* value of ≤ 0.05 were considered to be independent predictors of mortality.

### Ethical considerations and consent

All methods were carried out in accordance with relevant guidelines and regulations. Ethical approval was sought from the Research and Ethics Committee of Kampala International University Western Campus (Ref No: SF/202026). All participants were asked for written informed consent as evidenced by the participants’ signature or thumb prints of the legal guardian.

## Results

During the study period, 211 Victims of RTAs with a median age of 28 (IQR: 22–38) years were enrolled from the emergency units of the study centres. Majority of the participants were male (85.8%) aged 15–45 years (76.3%) with no comorbidity (95.3%). The most common road user category was motorcyclists accounting for 48.8% of the study participants. The rest of the details relating to the characteristics of study participants are shown in Table 1.

**Table 1:** Socio-demographic and clinical characteristics of the study participants

Characteristic	Frequency	Percent
**Age group**		
0–14	24	11.4
15–45	161	76.3
46 and above	26	12.3
**Sex**		
Male	181	85.8
Female	30	14.2
**Comorbidity**		
Cardiac disease	1	0.5
Diabetes	3	1.4
Hypertension	3	1.4
HIV	3	1.4
None	201	95.3
**Road user**		
Motorcyclist	103	48.8
vehicle driver	5	2.4
Motor cycle Passenger	30	14.2
Motor vehicle Passenger	3	1.4
Pedestrian	65	30.8
Bicyclist	5	2.4

*HIV* Human immunodeficiency virus

### 24 hour mortality

Of the 211 study participants, 31 died within 24 h from injury. Therefore the 24 h mortality was 14.69% (Table 2). Of the patients that died, 10 died due to Neurological sequelae, 1 hemorrhagic sequelae while majority [12] due to neurological + hemorrhagic sequelae.

**Table 2:** Incidence of 24 h mortality among study the participants

Category	Number of deaths	Percent
participants (*n* = 211)	31	14.69
Males (*n* = 181)	26	14.4
Females (*n* = 30)	05	16.7

### Predictors of 24 h mortality

At multivariate analysis, the only significant pre- and in-hospital predictors of mortality were road user category (*P* = 0.016) and injury severity (*P* < 0.001) respectively. According to our findings a pedestrian was 0.169 times less likely to die compared to a motorcyclist (aOR = 0.169, CI = 0.04–0.720, *P* = 0.016). This means that a motorcyclist was 5.917 (1/0.169) times more likely to die compared to a pedestrian. It was also observed that a patient who was categorized as having a moderate injury according to Kampala trauma score II was 0.064 times less likely to die compared to one that was categorized as having a severe injury (aOR = 0.064, CI = 0.019–0.210, *P* < 0.001) This means that a patient with severe injury was 15.625 (1/0.064) times more likely to die compared to one with a moderate injury. The details of bivariate and multivariate analysis are shown in Table 3.

**Table 3:** Pre-hospital and in-hospital predictors of 24 h mortality

		**Bivariate analysis**		**Multivariate analysis**	
**Variable**	**Number of deaths (%)**	**cRR (95%CI)**	***p*-value**	**aRR (95%CI)**	***p*-value**
**Pre-hospital**					
**Time to hospital in minutes**					
≤ 90	13 (41.9)	1.00			
> 90	18 (58.1)	1.23(0.573–2.679)	0.586		
**Age in yrs**					
≤ 14	4(12.9)	1.00			
15–45	25(80.6)	0.919(0.290–2.918)	0.886		
≥ 46	2(6.5)	0.417(0.069–2.516)	0.340		
**Mechanism of injury**					
Motor vehicle	21(67.7)	1.00		1.00	
Motorcycle	10 (32.3)	0.176(0.077–0.402)	<0.001	0.517(0.157–1.701)	0.277
Bicycle	0 (0.0)	N/A			
**Sex**					
Female	5 (16.1)	1.00			
Male	26 (83.9)	1.19(0.42–3.39)	0.74		
**Road user category**					
Motorcyclist	21 (67.7)	1.00			
Vehicle driver	0 (0.0)	N/A	0.999		
Motor cycle Passenger	2 (6.5)	0.252(0.056–1.138)	0.073	0.153(0.016–1.449)	0.102
Motor Vehicle Passenger	0 (0.0)	N/A			
**Pedestrian**	**8 (25.8)**	**0.548(0.227–1.323)**	**0.181**	**0.169(0.04–0.720)**	**0.016**
Bicyclist	0 (0.0)	N/A	0.999		
**In-hospital**					
**Time to First Aid**					
< 10.2 Minutes	22 (70.9)	1.00			
≥ 10.2 Minutes	9 (29.0)	0.643(0.280–1.4760	0.298		
**KTS II Severity score**					
Severe	23 (74.2)	1.00		1.00	
**Moderate**	**8 (25.8)**	**0.049(0.018–0.136)**	**< 0.001**	**0.064(0.019–0.210)**	**< 0.001**
Mild	0 (0.0)	N/A			
**Medical worker**					
Specialist	3 (9.7)	1.00			
SHO	11 (35.5)	2.01(0.492–8.211)	0.330		
Medical officer	7 (22.6)	0.60(0.140–2.572)	0.490		
Clinical officer	10 (32.3)	1.35(0.330–5.514)	0.680		
Nurse	0 (0.0)	N/A	0.999		
**Trauma training**					
Yes	14 (45.2)	1.00			
No	17 (54.8)	0.72(0.334–1.554)	0.403		
**Type of Injury**					
Blunt Injury	19 (61.3)	1.00		1.00	
Superficial Injury	6 (19.4)	0.654(0.235–1.819)	0.416	0.331(0.081–1.357)	0.124
Penetrating Injury	6 (19.4)	0.195(0.074–0.516)	0.001	0.541(0.154–1.903)	0.338

*CI* Confidence interval, *cOR* Crude odds ratio, *aOR* Adjusted odds ratio

## Discussion

Our findings reveal that the study participants were primarily young with a median age at 28 (IQR 22–38). The reported age of RTA victims varies by country, for example, 39 (IQR 27–52) years in India, 37.6 (± 15.4) years in Vietnam and 30 (± 13) years in Kenya [13–15]. Even though the exact median of the age reported varies, it's worth noting that, the median age was in the 30 s and the inter quartile ranges are overlapping showing that there is no significant difference in the age group injured in the different countries. The explanation for having this age group more commonly injured may be due to the fact that this age group has been ascribed to a variety of activities that require movement from one place to another. We also noticed that most of the study participants were males (85.8%) compared to the females (14.2%). This is consistent with findings from previous studies in Ugandan [16]. The majority of this group is more prone to be engaged in high-risk activities such as reckless riding, over-speeding, overloading, and riding while intoxicated and riding without any protective equipment which increases the risk of RTAs.

It was observed in this study that the most common road user category was motorcyclists accounting for 48.8% of the study participants. This is in accordance with a study by Chandrasekharan [17]. However, other studies showed different findings like Sisimwo et al. [15] and Kourouma et al. [18] who found pedestrians and passengers to be the most commonly injured. The large number of motorcyclists injured can be linked to the fact that motorcycles being two-wheeler have a lower stability compared to motor vehicles. The other possible reason could be because they have been reported to ignore most of the traffic rules that could keep them safe despite the low stability of the motorcycles [19]. Also, casualties among pedestrians (30.8%) could be explained by the lack of segregated pedestrian road networks and the lack of community understanding about road safety.

The incidence of early mortality in this study was relatively high (14.69%) and comparable with the LMICs’ rates as reported by WHO [2]. Tra et al. [14] in Korea observed also a high early mortality rate of 29.4% among road traffic accident victims. Huei et al. [20] and Kang et al. [12] reported a 10.8% and an 11.9% early mortality rate among patients with severe hemorrhagic shock due to trauma respectively. The differences seen in the mortality could be explained by the levels of care among other factors that may be study population or patient specific.

The only significant pre-hospital predictor of 24 h mortality was the category of road user, in which motorcycle riders were 5.9 times more likely to die compared to pedestrians. This is in agreement with the findings by Chandrasekharan [17], in which casualty fatalities were more common among motorcycle riders as compared to pedestrians. In contradiction to our findings, Derry, Palk and King [21] found that pedestrians had a 3 times higher relative risk of mortality than drivers or riders, and that being a driver or rider of any sort of vehicle was a protective factor against RTAs and fatalities when compared to being a pedestrian. The explanation to our findings is possibly because of the momentum of impact that increases the severity of injury among motorcycle riders who are likely to be at a higher speed compared to pedestrians.

In this study we used Kampala trauma score II (KTS II) to assess severity of injury and our findings showed that the severity of injury graded by KTS II was associated with 24 h mortality (*p* < 0.001). This is in line with earlier research, which suggested that severity of injury has a strong relationship with mortality rate [22, 23]. Weeks et al. [24] demonstrated in their study that the KTS II is as good as other grading systems at predicting patient mortality in resource limited settings.

Although studies have demonstrated type of injury (blunt and penetrating injuries) to be associated with mortality in trauma patients [20, 25] in this study, it was not statistically significant. The time to first aid, emergency unit workers and being trained on trauma care were also not significantly associated to early mortality in this study. The fact that we only assessed for 24 h mortality could explain the differences in our findings in relation to predictors since other studies did follow up for a longer period.

### Study limitations

Patients with minor injuries who did not seek treatment in the study sites were not identified. Also deaths that occurred on the scene or during transportation of the patient to hospital were not captured. In addition, follow-up stopped at 24 h and so deaths that occurred after 24 h were not captured.

## Conclusion and recommendations

The incidence of 24 h mortality among road traffic accident victims was high and being a motorcycle rider and severity of injury according to Kampala trauma score II predicted mortality. Therefore, motorcyclists should be reminded to be more careful while using the road. Trauma patients should be assessed for severity using KTS II, and the findings used to guide management.

We also recommend that a large non-hospital based cohort study of trauma patients be carried out and a follow-up of more than 24 h instituted in order to identify more predictors of mortality due to road accidents in our setting without excluding any patients.

## Data Availability

Data is available upon request. Requests should be sent to KK Via fistuskamabu@gmail.com.

## Abbreviations

KTS: Kampala trauma score
RTA: Road traffic accident
ATLS: Advanced trauma life support
KTS II: Kampala trauma score II

## References

[1] World_Health_Organization. Road traffic injuries. World Health Organization. 2022. p. 1. Available from: https://www.who.int/news-room/fact-sheets/detail/road-traffic-injuries. [Cited 6 Apr 2023].

[2] Groot K de. Global Status Report on Road Safety 2018- Un. WHO Press. Vol. 1, World Development. Geneva; 2018. 1–15 p. Available from: http://www.fao.org/3/I8739EN/i8739en.pdf%0A. 10.1016/j.adolescence.2017.01.003%0A. 10.1016/j.childyouth.2011.10.007%0A. 10.1080/23288604.2016.1224023%0A.

[3] Bawah A, Welaga P, Azongo DK, Wak G, Phillips JF, Oduro A (2014). Road traffic fatalities - a neglected epidemic in rural northern Ghana: evidence from the navrongo demographic surveillance system. Inj Epidemiol.

[4] Nations U, Commission E, Africa FOR, Nations U, Commission E, Europe for. United Nations Economic Commission For Africa Road Safety Performance Review Georgia. Layout and Printing at United Nations. New York and Geneva; 2018. Available from: http://www.unece.org

[5] Xinhua Africa. About Africa News: Uganda , Cameroon most affected by road traffic deaths , injuries in Africa : ECA. retrieved from http//www.xinhuanet.com/english/2019-05/07/c_138038561.htm. 2019;

[6] Hardcastle TC, Oteng R (2011). Trauma care in Africa: Triumphs and challenges. African J Emerg Med.

[7] Zia N, Mehmood A, Namaganda RH, Ssenyonjo H, Kobusingye O, Hyder AA (2019). Causes and outcomes of traumatic brain injuries in Uganda: analysis from a pilot hospital registry. Trauma Surg Acute Care Open.

[8] Daniel W. Biostatistics: A foundation for analysis in Health sciences. 7th Editio. Sons J wiley and, editor. New York; 1999.

[9] Oporia F, Kisakye AN, Nuwematsiko R, Bachani AM, Isunju JB, Halage AA (2018). An analysis of trends and distribution of the burden of road traffic injuries in Uganda, 2011 to 2015: a retrospective study. Pan Afr Med J.

[10] Dn K, Kiran A (2011). Emergency Trauma Care: ATLS. J Adv Oral Res.

[11] Weeks SR, Stevens KA, Haider AH, Efron DT, Haut ER, Mackenzie EJ (2016). A modified Kampala trauma score (KTS) effectively predicts mortality in trauma patients. Injury.

[12] Kang BH, Choi D, Cho J, Kwon J, Huh Y, Moon J (2017). Efficacy of uncross-matched type O packed red blood cell transfusion to traumatic shock patients: a propensity score match study. J Korean Med Sci.

[13] Gupta S, Khajanchi M, Kumar V, Raykar NP, Alkire BC, Roy N (2020). Third delay in traumatic brain injury: Time to management as a predictor of mortality. J Neurosurg.

[14] Tra T, Thao P, Khoi N, Hung L, Wada K, Brown F (2019). Clinical factors affecting mortality in patients with traumatic shock caused by road traffic accidents admitted to the emergency department: a prospective cohort study. Emerg Med Trauma Care J.

[15] Sisimwo PK, Mwaniki PK, Bii C (2014). Crash characteristics and injury patterns among commercial motorcycle users attending Kitale level IV district hospital. Kenya Pan Afr Med J.

[16] Balikuddembe J, Ardalan A, Zavareh D, Nejati A, Munanura K (2017). Road traffic incidents in Uganda: A systematic review study of five years trend. J Inj Violence Res.

[17] Chandrasekharan A, Nanavati AJ, Prabhakar S, Prabhakar S (2016). Factors impacting mortality in the pre-hospital period after road traffic accidents in Urban India. Trauma Mon.

[18] Kourouma K, Delamou A, Lamah L, Camara BS, Kolie D, Sidibé S (2019). Frequency, characteristics and hospital outcomes of road traffic accidents and their victims in Guinea: A three-year retrospective study from 2015 to 2017. BMC Public Health.

[19] Olasinde AA, Oluwadiya KS, Sikakulya FK, Muhumza J (2022). Road safety regulations : how compliant are commercial motorcyclists in Semi-Urban towns in Western Nigeria?. East African J Heal Sci.

[20] Huei TJ, Mohamad Y, Lip HTC, Noh NM, Alwi RI (2017). Prognostic predictors of early mortality from exsanguination in adult trauma: a Malaysian trauma center experience. Trauma Surg Acute Care Open.

[21] Damsere-Derry J, Palk G, King M (2017). Road accident fatality risks for “vulnerable” versus “protected” road users in northern Ghana. Traffic Inj Prev.

[22] Chalya PL, Mabula JB, Dass RM, Mbelenge N, Ngayomela IH, Chandika AB (2012). Injury characteristics and outcome of road traffic crash victims at Bugando medical centre in Northwestern Tanzania. J Trauma Manag Outcomes.

[23] Seid M, Azazh A, Enquselassie F, Yisma E (2015). Injury characteristics and outcome of road traffic accident among victims at adult emergency department of Tikur Anbessa specialized hospital, Addis Ababa, Ethiopia: a prospective hospital based study. BMC Emerg Med.

[24] Weeks SR, Juillard CJ, Monono ME, Etoundi GA, Ngamby MK, Hyder AA (2014). Is the Kampala Trauma Score an effective predictor of mortality in low-resource settings? a comparison of multiple trauma severity scores. World J Surg.

[25] Gönültaş F, Kutlutürk K, Gök AFK, Barut B, Şahin TT, Yılmaz S (2020). Analysis of risk factors of mortality in abdominal trauma. Ulus Travma ve Acil Cerrahi Derg.

